# Low Back Pain among Healthcare Workers in University Hospital in Tunisia

**DOI:** 10.2174/0115733971387161250916043838

**Published:** 2025-10-01

**Authors:** Noura Belhadj, Ines Rassas, Cyrine Ben Taleb, Narjes Belhadj Chabbah, Asma Gaddour, Asma Kheder, Aouatef Mahfoudh

**Affiliations:** 1 Laboratory of Ergonomic Management of Occupational and Environmental Risks, Faculty of Medicine of Monastir, University of Monastir, Monastir, Tunisia;; 2 Occupational Medicine Department, Taher Sfar University Hospital, Mahdia, Tunisia;; 3 Occupational Medicine Department, Sahloul University Hospital, Sahloul, Tunisia;; 4 Occupational Medicine Department, Ibn Eljazzar University Hospital, Kairouan, Tunisia

**Keywords:** Health professional, healthcare, low back pain, degeneration, risk factors, occupational health

## Abstract

**Introduction:**

Low back pain is a real public health issue. It is a common musculoskeletal disorder linked to work among health professionals. The objective of this study is to determine the frequency of low back pain among healthcare workers and to study the associated risk factors.

**Methods:**

This is a cross-sectional study conducted over a period of 6 months during the year 2022 involving a sample of the healthcare staff at Mahdia University Hospital. An anonymous self-administered questionnaire was completed by the healthcare staff, including anthropometric, socio-economic, professional lifestyle habits, and the characteristics of low back pain.

**Results:**

A total of 96 participants responded to the questionnaire. The population was mostly female (75.3%) with an average age of 36.21 ± 8.78 years. The average BMI was 27.7 kg/m^2^ ± 4.5 kg/m^2^, with nurses being the most numerous group, followed by midwives. The professional activities were mainly care activities (76.7%). The frequency of low back pain was estimated at 82.2% (n=79). In univariate analysis, a significant association was observed between the low back pain and age (*p* < 0.001), marital status (*p* = 0.027), physical activity (*p* = 0.03), job seniority (*p* = 0.001), care activities (*p* = 0.03), sitting position (*p* = 0.04) and weight carried (*p* = 0.003).

**Discussion:**

The prevalence of low back pain in our study was 82.2%. This finding aligns with results from studies conducted in Egypt at Zigazig Hospital (79%) and in Rwanda at Kanombe Military Hospital (78%).

**Conclusion:**

In this study, multiple factors linked to low back pain were identified, most of which are modifiable, highlighting the need to implement effective preventive measures to reduce the prevalence of low back pain and limit the socio-economic damage it generates.

## 1 INTRODUCTION

Low back pain (LBP) is a common symptom that is defined by a feeling of lower back pain unrelated to a traumatic, inflammatory, infectious or tumor cause [1, 2]. The diagnosis is then made in the absence of a cause that can explain the pain apart from degeneration [3].

Common low back pain is now the second pain felt after headaches, and the leading cause of osteoarticular morbidity [2, 3].

This pathology, whose prevalence continues to increase, affects people of all ages. This is explained by the existence of multiple risk factors linked to everyday life and work. Therefore, in France, in a hard-working environment, more than 2 out of 3 employees have had lower back pain. LBP represents 20% of work accidents and 7% of occupational diseases [4].

Several studies have evaluated the prevalence of back pain among healthcare workers. A recent systematic review determined that the global prevalence of low back pain among nurses ranged from 50% to 80% [5].

In some countries, common low back pain can be classified as an occupational disease with well-respected conditions [6]. It affects specific groups of workers who experience excessive strain on the lower back [3].

It is currently the leading cause of disability and incapacity to work in the world [1, 6]. In Tunisia, the prevalence of low back pain was estimated at 51.7% among nurses and 57.1% among all hospital staff [7].

This generates a serious medical and socio-economic cost, especially for low- or middle-income countries where the possibility of changing workstations is almost absent [6].

Taking into account the nature of the work of health professionals, with the dynamic and static loads that they undergo in the long term, they are the first to be prone to suffering from LBP [4, 8].

It is then reasonable to determine the frequency of low back pain among healthcare workers and to identify the associated risk factors, not only professional but also those linked to the lifestyle of healthcare providers, such as smoking and physical activity.

## 2 METHODS

### 2.1 Participants

An analytical cross-sectional study was conducted over a period of 6 months from January 2022 to June 2022. The study included healthcare personnel working at Mahdia University Hospital, including nurses, midwives, workers, nursing assistants, emergency technicians and physiotherapists, who had worked in the post for at least 1 year and had given their consent to participate in the study.

### 2.2 Study Size

The calculation of the minimum sample size was based on the formula below. n = t^2^ × p × (1-p)/m^2^

n: Minimum sample size to obtain significant results

t: Confidence level (the typical value of the 95% confidence level will be 1.96)

p: Estimated prevalence of chronic low back pain among nurses according to the study by Budhrani-Shani P. *et al*. carried out in 2016 [5]

m: Margin of error (set at 10%)

Taking these values into account makes it possible to determine the minimum sample size: n=1.96^2^*0.5*(1-0.5) / 0.1^2^= 96

### 2.3 Questionnaire

Data collection was based on a questionnaire that consisted of five sections:

-Anthropometric characteristics (sex, age, height, weight, BMI).
-Lifestyle habits such as smoking, physical exercise, household activities, mode of transport, mattress hardness, sleeping position, *etc*.
-Socioeconomic parameters (level of study, socioeconomic level, marital status, number of births).
-Professional characteristics (position held, professional seniority, working hours, work postures, handling operations carried out and their weight, physical load, *etc*).
-Characteristics and medical information about the low back pain: the age of onset of pain, the severity of the pain, its impact on activity and the therapeutic means used (medical, orthopedic or surgical).

To assess back problems, we opted for the Nordic questionnaire [9]. It is a sensitive and useful instrument in the screening and monitoring of musculoskeletal disorders. In the present study, we are only interested in items dedicated to the back.

### 2.4 Statistical Analysis

Analyses were carried out using the Statistical Package for the Social Sciences (SPSS) statistics program version 21. Chi-2 and Fisher exact tests were performed to test for differences in proportions of categorical variables between the two groups: presence of low back pain and absence of low back pain.

Multivariable logistic regression was used to predict factors that influence the presence of low back pain. In this model, only significant variables of interest at a threshold of 20% (*p* < 0.2) were introduced. The level *p* < 0.05 was considered as the significance threshold.

## 3 RESULTS

The present study included 96 healthcare workers out of a total of 116, representing a participation rate of 82.7%. The average age of our sample was 36.2 ± 8.7 years with a clear female predominance (75.3%). The mean BMI was 27.7 ± 4.5 kg/m^2^ with ranges of 20.4 and 43.2 kg/m^2^. Among the participants, 50.4% had a higher level of education and 35.6% had a secondary level of education. The majority of participants (60%) were married. The mean number of pregnancies was 2.6 ± 1.5. Regular tobacco consumption was reported in 12.3% of participants.

Regular sports activity was noted in 11% of healthcare workers. Thirty-four percent of participants performed household activities regularly.

The most common means of transport was their personal car. The hardness of the mattress was average in 50.7% of cases. The most commonly described sleeping position was on the side at 61.6%.

The distribution of participants in the labor departments was varied (Table **1**). Among the participating staff, nurses were the most numerous, followed by midwives. The professional activities were mainly care activities (76.7%). The average job seniority of the participants was 12 ± 9 years (2 -25 years), and 61.4% of them had a seniority of less than 10 years.

The standing position was reported in 61.6% of cases with a time interval between 6 and 8 hours (Table **2**). The forward-leaning posture was noted in 34.2% of cases for 30 min to 2 hours per day.

Fifty-six percent of participants reported carrying heavy loads with a weight greater than 20 kg.

The time spent carrying loads per day was between 0 and 30 min in 36.5% of cases, with a duration of carrying that did not exceed 5 min in 32.9% of cases (Table **2**).

Table **3** details all the answers to the Nordic questionnaire. The frequency of low back pain was estimated at 82.2% (n = 79) at least once during their life and at 93.3% during the last 12 months. Low back pain was stabbing in 33% of participants, and of intermediate severity in 41.7% of cases.

Sixty-seven percent of participants sought medical treatment. And 61.7% benefited from sick leave because of their low back pain lasting between 1 and 7 days in 46.7% of cases.

Comparison between individuals with and without low back pain showed that only age (*p* ≤ 10^-3^), marital status (*p* = 0.02), and physical activity (*p* = 0.03) were significantly associated with low back pain (Table **4**).

Regarding professional factors, low back pain was significantly associated with job seniority (*p* = 0.001), care activities (*p* = 0.03), sitting position (*p* = 0.04), and weight carried (*p* = 0.003) (Table **4**).

In the multivariate analysis, the presence of LBP was multiplied by a factor of 2.2 among the age group (> = 30 ans) (*p* ≤ 10^-3^; OR=2.2; 95% CI = (1.3; 3.3)) and by a factor of 3.4 among care activity (*p* = 0.03; OR=3.4; 95% CI = (1.2; 7.4)) (Table **5**).

## 4 DISCUSSION

Low back pain is a common work-related musculoskeletal disorder among healthcare workers, particularly nurses, in a hospital environment [8, 10].

Several studies have shown that health care providers are most likely to have low back pain compared to other professional groups and the general population [11-13].

Given that it is responsible for psychophysical discomfort, low back pain hinders the functioning of daily life, reduces the quality of life, and can, in turn, alter the quality of care presented to patients [8, 14].

The main objective of this study was to determine the frequency and associated risk factors.

The frequency of low back pain in our study was 82.2%. This result is consistent with those found in studies carried out in Egypt, at the Zigazig hospital [15] (79%), in Rwanda, at the Kanombe military hospital (78%) [16], and at the Nigerian hospital (73%) [17].

Similarly, the study by Kalabalik *et al*. in Türkiye concluded that 56.5% of all participants, 59.9% of nurses, and 51.4% of caregivers reported having suffered from low back pain in the past month [18].

However, these results are significantly higher than those recorded in Addis Ababa, Ethiopia (45.8%) [19] and Ibadan, Nigeria (44.1%) [20]. This study showed that low back pain is higher among female caregivers (75.3%) with no significant association. In Tunisia, in the study by Bejia *et al*., female gender was a risk factor associated with common low back pain (*p* = 0.02) [7].

This could be explained by the anatomical and structural differences between men and women [21, 22]. Physiological conditions such as pregnancy and menstruation could also explain this difference [23, 24].

In our study, nursing staff who were older than 30 years had the highest frequency of low back pain with a significant association (*p* < 0.001). However, the work of Belay *et al*. revealed a high prevalence in people whose age is less than 30 years (60.2%) [19].

This could be explained by the frequent comorbidities in this age group and the physical load they undergo during years of exercise [25].

In the present study, 91% of married women had low back pain with a significant association equal to 0.027. The results of the studies on the relationship between marital status and LBP available in the literature are contradictory. Some studies reported that LBP was more common among married hospital employees [7, 15], whereas others did not find a significant relationship between marital status and LBP [18, 26].

This may be due to the responsibility and family burden carried by women, especially in our Tunisian context, where family members are predominantly numerous. However, according to the studies of Moussa *et al*. and Heutink *et al*., pregnancy constitutes a risk factor associated with low back pain [27].

In our study, we found a significant association between low back pain and the absence of physical activity (*p* = 0.03). According to the study of Citko *et al*., a sedentary lifestyle increases the risk of low back pain among healthcare professionals by 3.5 [3].

Occupational risk factors play an important role in the development of LBP. Some of the occupational risk factors mentioned in the literature include lifting patients or heavy subjects without using a lifting device, transferring patients alone, and remaining in the same position for extended periods [28].

A study in Uganda found that staff responsible for direct patient care have the highest prevalence of LBP, since they are most involved in manual handling of patients [25].

Sikuri *et al*. found that manual handling of loads in a Nigerian hospital is a serious occupational risk factor associated with LBP [17].

In our study, the professional activities most responsible for LBP were office and household activities with a statistically significant association (*p* = 0.003). This could be explained by prolonged sitting, the resulting weight gain and lack of awareness of prevention strategies in the workplace. In parallel, Kalabalik stated that the frequency of LBP was found to be significantly higher among those who stand for extended periods (*p* = 0.024) [18].

In this study, personnel carrying loads of less than 15 kilos suffered the most from low back pain with a significant association *p* = 0.03. This could be explained by the fact that the staff most responsible for carrying heavy loads have acquired a certain experience and knowledge in ergonomics, and that the hospital structure has not yet managed to provide equipment for automatic handling systems for lighter loads [29].

In our study, prolonged sitting (≥ 4 hours) was a risk factor associated with low back pain (*p* ≤ 10^-3^). Hussain *et al*. found that in an Australian population of adults, watching television for 2 hours per day can cause low back pain [30].

Like any work, our study has certain limitations that deserve mention.

Firstly, being a cross-sectional study, it can only provide associations and not causal relationships.

Furthermore, a reporting bias may be posed by our study. To overcome this bias, we recommended at the questionnaire level a preamble recalling the purpose of the study while insisting on respect for confidentiality and anonymity.

## CONCLUSION

Low back pain is a frequent reason for consultation and a widespread symptom in all adult populations. It is a common and important occupational health concern among healthcare workers.

This study shows the importance of individual and professional factors in the occurrence of low back pain. The univariate study showed that the age group over 30 years and married patients have a higher risk of low back pain. Meanwhile, the professional factors associated with low back pain were caregiving activities, lifting weights over 15 kilos, and sitting for more than 4 hours per day.

In this study, following multivariate analysis, the predictive factors for low back pain (LBP) were identified as: age group (≥30 years) (*p* ≤ 0.001; OR=2.2; 95% CI = (1.3, 3.3)) and caregiving activity (*p* = 0.03; OR=3.4; 95% CI = (1.2, 7.4)).

Manual patient handling, long periods of standing, and repetitive movements are just a few of the physically demanding tasks that nurses must do on a daily basis. In order to effectively manage lower back pain (LBP), it is essential to have a thorough plan that encourages physical health, proper work organization, and continuous education and training.

For this, several recommendations were proposed, such as the implementation of automatic assistance for handling, the application of regular training on ergonomics and healthy living for all healthcare staff. Moreover, we must encourage collaboration between nurses, physiotherapists, and other healthcare professionals to develop comprehensive and individualized rehabilitation and self-management programs for nurses suffering from low back pain, taking into account their training

Also, professional adaptation for people with low back pain may limit the risk of becoming chronic and adapting work hours in order to have good quality sleep and a balance between family and professional life.

Additional studies are necessary to better study the direct and indirect repercussions of low back pain to highlight its seriousness and, therefore, the importance of implementing preventive measures.

## Figures and Tables

**Table 1:** Occupational characteristics of the study population.

**-**	**Number (N)**	**Percentage (%)**
**Work sector:** Obstetric gynecology Urgences Endocrinolgy Anesthesia-intensive Surgery Urology Neonatology	18 18 16 14 12 10 8	18.75 18.75 16.7 14.6 12.5 10.4 8.3
**Professional category:** Nurses Midwives Workers Others (Emergency Technicians, Caregivers…)	59 8 17 12	61.5 8.3 17.7 12.5
**Job tenure** ≤ 10 years >10 years	59 37	61.5 38.5
**Pace of work** Day work Night work Shift work	47 23 26	49 24 27

**Table 2:** Distribution of participants according to the duration of standing position and of carrying heavy loads.

**-**	**Number (N)**	**Percentage (%)**
**Duration of standing** 0-30 minutes 2-4 hours 4-6 hours 6-8 hours	- 5 10 8 73	- 5.2 10.4 8.3 76.1
**Duration of carrying heavy loads** 0-30 mins 30 mins-2 hours 2-4 hours 4-6 hours 6-8 hours	- 35 29 13 8 11	- 36.5 30.2 13.5 8.3 11.5

**Table 3:** Standardised nordic questionnaire.

**-**	**Number (N)**	**Percentage (%)**
**During life**	-	-
*Experienced lower back problems*	-	-
***Yes***	79	82.2
***No***	17	17.8
*Lower back injury in an accident*	-	-
***Yes***	20	20.8
***No***	76	79.2
*Change of job or task due to lower back problems*	-	-
***Yes***	18	18.7
***No***	78	81.3
**In the last 12 months**	-	-
*Experienced lower back problems*	-	-
**Yes**	89	93.3
**No**	7	6.7
*Total duration of lower back problems*	-	-
**1-7 days**	60	62.6
**8-30 days**	18	18.7
**>30 days**	18	18.7
*Reduction in activities due to low back pain*	-	-
**Usual activities at work and/or at home**	-	-
**Yes**	61	63.3
**No**	35	36.7
**Hobbies**	-	-
**Yes**	37	38.3
**No**	59	61.7
*Total duration of reduction in usual activities due to low back pain*	-	-
**0 days**	13	13.3
**1-7 days**	49	51.7
**8-30 days**	18	18.3
**>30 days**	16	16.7
*Consultation with a health professional for back problems*	-	-
**Yes**	47	48.3
**No**	49	51.7
**In the last 7 days**	-	-
*Feeling discomfort in the lower back*	-	-
**Yes**	52	53.3
**No**	44	46.7

**Table 4:** Individual and professional factors associated with the low back pain.

-	Low Back Pain(+) N(%)	Low Back Pain(-) N(%)	*p*
**Gender:** **Male** **Female**	19(24) 60(79)	5(29.4) 12(70.6)	0.07
**Age group:** >=30 ans <30 ans	53(67) 26(33)	8(47) 9(53)	≤10^-3^
**Marital status:** Married others	52(65.8) 27(34.2)	5(29.4) 12(70.6)	0.02
**pregnancies** yes no	58(73.4) 21(26.6)	10(58.8) 7(41.2)	0.1
**Tobacco smoking** Yes no	25(31.6) 54(68.4)	11(64.7) 6(35.3)	0.19
**Physical activity** Yes No	43(54.4) 36(45.6)	13(76.4) 4(23.6)	0.03
**Job tenure** ≤10 years >10 years	51(64.5) 28(35.5)	8(47) 9(53)	0.1
**Professional activities:** -Care activity -Office and household activity	61(77.2) 18(22.8)	12(15.2) 5(84.8)	0.03
**The weight carried** <15 kilos >= 15 kilos	36(45.6) 43(54.4)	4(23.5) 13(76.5)	0.003
**Standing position** <2h >=2h	26(32.9) 53(67.1)	4(23.5) 13(76.5)	0,3
**Sitting position** < **4 hours** >=**4 hours**	37(46.8) 42(53.2)	3(17.6) 14(82.4)	≤10-3

**Note:** The *p*-value reported refers to the statistical significance of the results based on chi-square test. The *p*-value is a measure of the probability that the observed results occurred by chance, with values typically below 0.05 indicating statistical significance.

**Table 5:** Predictive factors for low back pain.

**-**	**Low Back Pain(+)**	**Low Back Pain(-)**	***p***	**OR**	**95% CI**
**Age group:** >=30 ans <30 ans	53(67) 26(33)	8(47) 9(53)	≤10^-3^	2.2	(1.3; 3.3)
**Marital status:** Married others	52(65.8) 27(34.2)	5(29.4) 12(70.6)	0.02	0.5	(0.09; 1.8)
**Physical activity** Yes No	43(54.4) 36(45.6)	13(76.4) 4(23.6)0	0.03	0.04	(0.01; 7.8)
**Professional activities:** **-**Care activity -Office and household activity	61(77.2) 18(22.8)	12(15.2) 5(84.8)	0.03	3.4	(1.2; 7.4)
**The weight carried** **<**15 kilos >= 15 kilos	43(54.4) 36(45.6)	4(23.5) 13(76.5)	0.003	0.4	(0.01; 7.8)
**Sitting position** **< 4 hours** **>=4 hours**	37(46.8) 42(53.2)	3(17.6) 14(82.4)	≤10-3	4.2	(0.01; 7.8)

## Data Availability

The data and supportive information are available within the article.

## LIST OF ABBREVIATIONS

LBP: Low Back Pain
SPSS: Statistical Package for the Social Sciences

## STANDARDS OF REPORTING

STROBE guidelines were followed.
